# Genomic epidemiology of multidrug-resistant and non-multidrug-resistant clinical *Salmonella enterica* isolates in Guangdong, China

**DOI:** 10.3389/fcimb.2026.1852160

**Published:** 2026-10-23

**Authors:** Sitong Chen, Jiajun Liu, Dongmei He, Bojun Wang, Ouyang Fangzhu, Yahui Shi, Bixia Ke, Baisheng Li

**Affiliations:** 1 Guangdong Provincial Center for Disease Control and Prevention, Guangzhou, China; 2 School of Public Health, Guangdong Pharmaceutical University, Guangzhou, China; 3 Guangdong Provincial Key Laboratory of Pathogen Detection for Emerging Infectious Disease Response, Guangzhou, China; 4 Guangdong Workstation for Emerging Infectious Disease Control and Prevention, Chinese Academy of Medical Sciences, Guangzhou, China; 5 School of Public Health, Southern Medical University, Guangzhou, China

**Keywords:** clinical isolates, genomic epidemiology, multidrug resistance, population structure, *Salmonella enterica*, whole-genome sequencing

## Abstract

*Salmonella enterica* (*S. enterica*) is a major foodborne pathogen that threatens human health. Multidrug resistance (MDR) has complicated the prevention and treatment of salmonellosis. The present study aimed to characterize the antimicrobial resistance patterns, population structure, and genomic features of clinical *S. enterica* isolates from Guangdong, China. Among 4413 isolates collected between 2022 and 2024, 1084 underwent antimicrobial susceptibility testing and 170 were further analyzed by whole-genome sequencing. MDR was defined as resistance to at least three antimicrobial classes and was identified in 71.13% of the tested isolates. Genomic analyses included serotyping, multilocus sequence typing, resistance and virulence gene analyses, and core-genome phylogenetic analysis. XGBoost and LASSO were used to prioritize genomic features associated with MDR phenotype. Serovar I 4,[5],12:i:- was the predominant serovar among MDR isolates, while ST34 and ST19 were the most common sequence types. Compared with non-MDR isolates, MDR isolates more frequently carried several resistance genes, including *CTX-M-55*, *qnrS1*, *tet(A)*, *floR*, *sul2*, *sul3*, and *TEM-1.* Differences in virulence gene content were also observed between the two groups. Both XGBoost and LASSO consistently prioritized *floR* and *CTX-M-55* as candidate genomic features associated with the MDR phenotype. Overall, MDR and non-MDR clinical *S. enterica* isolates from Guangdong differed markedly in lineage distribution and gene carriage, providing a genomic basis for regional surveillance of MDR *S. enterica*.

## 1 Introduction

*Salmonella enterica* (S. enterica) is a major foodborne pathogen and a leading cause of diarrheal disease worldwide. Non-typhoidal *Salmonella* causes an estimated 93.8 million cases of gastroenteritis and 155,000 deaths each year globally (Majowicz et al., 2010). Most infections are self-limiting and present as gastroenteritis (Lamichhane et al., 2024). However, some cases can develop into invasive disease, such as bacteraemia or meningitis, especially in young children, older adults, and immunocompromised patients (Fierer, 2022). These severe infections often require antimicrobial treatment and may lead to life-threatening complications (Marchello et al., 2022).

Antimicrobial resistance has become a major challenge in the control of *Salmonella* infections (Sati et al., 2025; Lu et al., 2024). Multidrug-resistant isolates are of particular concern. Resistance to fluoroquinolones and third-generation cephalosporins is also worrying. These drugs remain important for severe *Salmonella* infections (Yu et al., 2024). Recent studies from China have shown the same trend. In Hangzhou, resistance to ciprofloxacin and cefotaxime reached 19.19% and 16.24%, respectively (Yu et al., 2024). The MDR rate among outpatient isolates was 56.6% in Shanghai (Lv et al., 2025). In Chongqing, 59.10% of isolates from diarrheal patients were multidrug resistant (Kong et al., 2025). A nationwide study also reported cefotaxime resistance of 25.91% and an MDR rate of 87.98% in human *S.* I 4,[5],12:i:- isolates (Sun et al., 2025). These findings show that AMR (antimicrobial resistance) is widespread in China.

Many studies in China are still descriptive and mainly focus on serovars, antimicrobial resistance, or specific serovars (Cao et al., 2025; Ma et al., 2024). In Guangdong, existing genomic studies have mainly examined specific serovars or non-clinical sources, such as sewage isolates (Sun et al., 2024; Su et al., 2024). Few studies have directly compared MDR and non-MDR clinical isolates at the genomic level (Luo et al., 2022; Mai et al., 2025; Wang et al., 2023). A clearer genomic picture of clinical *S.* enterica isolates is still needed in Guangdong.

Conventional antimicrobial susceptibility testing can show whether an isolate is resistant, but it cannot explain the underlying genetic basis (McDermott et al., 2016; Ellington et al., 2017; Cooper et al., 2020). Whole-genome sequencing (WGS) provides detailed information on resistance genes, virulence genes, and population structure (Lv et al., 2025; Zhuo et al., 2024). It also allows direct comparison between MDR and non-MDR isolates at the genome level (Go et al., 2025). Such comparisons may help identify genomic features linked to the MDR phenotype (He et al., 2025; Woh et al., 2025). Because WGS generates high-dimensional data, machine learning approaches may help prioritize important genetic features associated with MDR (Nguyen et al., 2019; Ayoola et al., 2024).

In this study, we analyzed clinical *S.* enterica isolates collected in Guangdong Province, China, from 2022 to 2024. We combined antimicrobial susceptibility testing with whole-genome sequencing to characterize their resistance profiles, population structure, and genomic features. We then compared MDR and non-MDR isolates to identify genomic differences associated with the MDR phenotype. Finally, we used XGBoost and LASSO to screen important genetic features related to MDR.

## 2 Materials and methods

### 2.1 Ethics statement

This study was approved by the Ethics Committee of the Guangdong Provincial Center for Disease Control and Prevention (No. W96-027E-202201). All procedures followed the approved ethical guidelines. Informed consent was obtained from all patients or their legal guardians during sample collection. All patient data were anonymized before analysis.

### 2.2 Collection, isolation and identification of bacterial isolates

During the Guangdong Provincial Foodborne Disease Risk Surveillance Program from 2022 to 2024, a total of 4413 Salmonella isolates were collected from clinical specimens obtained from patients at 31 sentinel hospitals and municipal Centers for Disease Control and Prevention across 14 cities in Guangdong Province, China. Among these isolates, 1084 available isolates were selected for antimicrobial susceptibility testing (AST). Of these, 170 isolates were further selected for whole-genome sequencing (WGS) based on isolate availability, DNA quality, and sequencing feasibility, while retaining both MDR and non-MDR isolates for comparative genomic analysis. Detailed information on the 170 sequenced isolates is provided in **Supplementary Table S2**.

Isolation and identification of Salmonella were performed according to the Foodborne Disease Surveillance Manual (Version 2022) issued by the China National Center for Food Safety Risk Assessment (CFSA). Briefly, stool specimens or anal swabs were collected from suspected foodborne illness cases. Fresh stool samples were placed in sterile dry containers and transported to the laboratory under cold-chain conditions within 8 h. Specimens stored in Cary–Blair transport medium were kept at 4 °C and processed within 24 h.

Samples were inoculated into SBG enrichment broth and incubated at 36 °C for 18–24 h. After enrichment, one loopful of broth was streaked onto *Salmonella* chromogenic agar or XLD agar plates and incubated at 36 ± 1 °C for 18–24 h. Three to five suspected colonies were selected for species identification by matrix-assisted laser desorption/ionization time-of-flight mass spectrometry (MALDI-TOF MS; bioMérieux, Lyon, France). Serovars were determined using specific O and H antisera (Statens Serum Institut, SSI) according to the White–Kauffmann–Le Minor scheme (Grimont and Weill, 2007). For WGS-analyzed isolates, serovars were predicted in silico using SeqSero2 (v1.2.1) in genome-assembly mode (Zhang et al., 2019). For consistency with the serovar analysis of the full set of 1084 isolates, phenotypic serovar assignments were used for the serovar-based analyses, tables, and phylogenetic tree annotations, whereas SeqSero2 predictions were retained as an independent genomic comparison and are reported in **Supplementary Table S2**.

### 2.3 Antimicrobial susceptibility testing

Antimicrobial susceptibility testing was performed by broth microdilution for 13 antimicrobial agents: ciprofloxacin (CIP), ampicillin (AMP), ampicillin/sulbactam (SAM), ceftazidime (CAZ), cefotaxime (CTX), cefoxitin (FOX), cefazolin (CZO), tetracycline (TCY), imipenem (IPM), polymyxin B (POL), gentamicin (GEN), chloramphenicol (CHL), and trimethoprim-sulfamethoxazole (SXT). Minimum inhibitory concentrations (MICs) were interpreted according to the Clinical and Laboratory Standards Institute M100, 31st edition (CLSI, 2021). *Escherichia coli* ATCC 25922 was used as the quality control strain. Isolates were identified as MDR if they were resistant to ≥3 antibiotic classes (Magiorakos et al., 2012).

### 2.4 Whole-genome sequencing

Genomic DNA was extracted from the 170 selected *Salmonella* isolates using the Magen HiPure Bacterial DNA Kit according to the manufacturer’s instructions. Then, high-throughput whole-genome sequencing (WGS) was performed using the Illumina HiSeq 2000 platform (Illumina, https://www.illumina.com), obtaining paired-end 150-bp reads. The quality of the raw sequenced reads was assessed using FastQC version 0.11.9 (Andrews, 2010). The raw reads were assembled using SPAdes version 3.6.1 (Bankevich et al., 2012), with read error correction enabled. Genome assembly quality was assessed using QUAST (v5.2.0) (Gurevich et al., 2013). The assembly statistics, including genome size, GC content, number of contigs, N50 and L50 values, and mean coverage, were summarized for all sequenced isolates and are provided in **Supplementary Table S1**. Assemblies were considered acceptable when the mean coverage was ≥20×, the N50 value was ≥50 kb, and the number of contigs was ≤500. Genome size, GC content, and L50 were additionally examined as quality indicators. Assemblies not meeting these quality criteria were excluded from downstream genomic analyses. The pubMLST database of *Salmonella* (Jolley et al., 2018) was used to assign the sequence type (ST) of each isolate based on seven housekeeping genes (*aroC, dnaN, hemD, hisD, purE, sucA*, and *thrA).* Antimicrobial resistance genes were identified using the Resistance Gene Identifier (RGI) against the Comprehensive Antibiotic Resistance Database (CARD) (Alcock et al., 2023), and virulence genes were identified using the Virulence Factor Database (VFDB) (Liu et al., 2022). A core-genome phylogenetic tree was constructed based on the core genome alignment generated by Panaroo (v1.8.0) (Tonkin-Hill et al., 2020). Maximum-likelihood phylogenetic analysis was performed using IQ-TREE (v3.1.2) (Wong et al., 2026). The best-fit nucleotide substitution model was selected by ModelFinder (Kalyaanamoorthy et al., 2017) based on the Bayesian information criterion (BIC), and the GTR+F+R7 model was applied for tree reconstruction. Branch support was assessed using the ultrafast bootstrap method with 1000 replicates (Hoang et al., 2018). The resulting maximum-likelihood tree was midpoint-rooted before visualization and annotation using ChiPlot (ChiPlot).

### 2.5 Statistical analysis

Comparisons of antimicrobial resistance phenotypes, resistance genes, virulence factors, and sequence types (STs) between MDR and non-MDR isolates were performed using Pearson’s chi-squared test or Fisher’s exact test when the expected cell count was <5. Statistical significance was defined as a two-tailed P < 0.05. Statistical analyses were performed using Stata V16.0 (StataCorp LP, College Station, Texas, USA). To account for potential confounding by clonal population structure, resistance gene–MDR associations were additionally assessed using the Cochran–Mantel–Haenszel test stratified by sequence type, with *P* values adjusted using the Benjamini–Hochberg method.

MDR status was treated as a binary outcome, and the presence or absence of antimicrobial resistance and virulence genes was used as candidate variables. LASSO logistic regression was performed in R using the “glmnet” package with a binomial distribution and an alpha value of 1 (Friedman et al., 2010). The penalty parameter λ was selected by cross-validation, and genes with non-zero coefficients at λmin were retained. XGBoost was then applied to the same candidate variables using the “xgboost” package in R (Chen and Guestrin, 2016). The model was fitted with 1000 boosting rounds, a maximum tree depth of 3, and a learning rate of 0.01. Feature importance was evaluated using the Gain metric, and genes identified by both methods were selected for further interpretation.

## 3 Results

### 3.1 Bacteria isolation

From January 1, 2022, to December 31, 2024, a total of 4413 *Salmonella* isolates were recovered. The isolates showed a clear seasonal pattern, with higher numbers between May and October. The highest detection rates occurred consistently in June each year (36.00% in 2022, 40.88% in 2023, and 31.21% in 2024), whereas the lowest rates were observed in December (6.70%, 11.23%, and 6.85%, respectively) (**Figure 1**).

**Figure 1:**
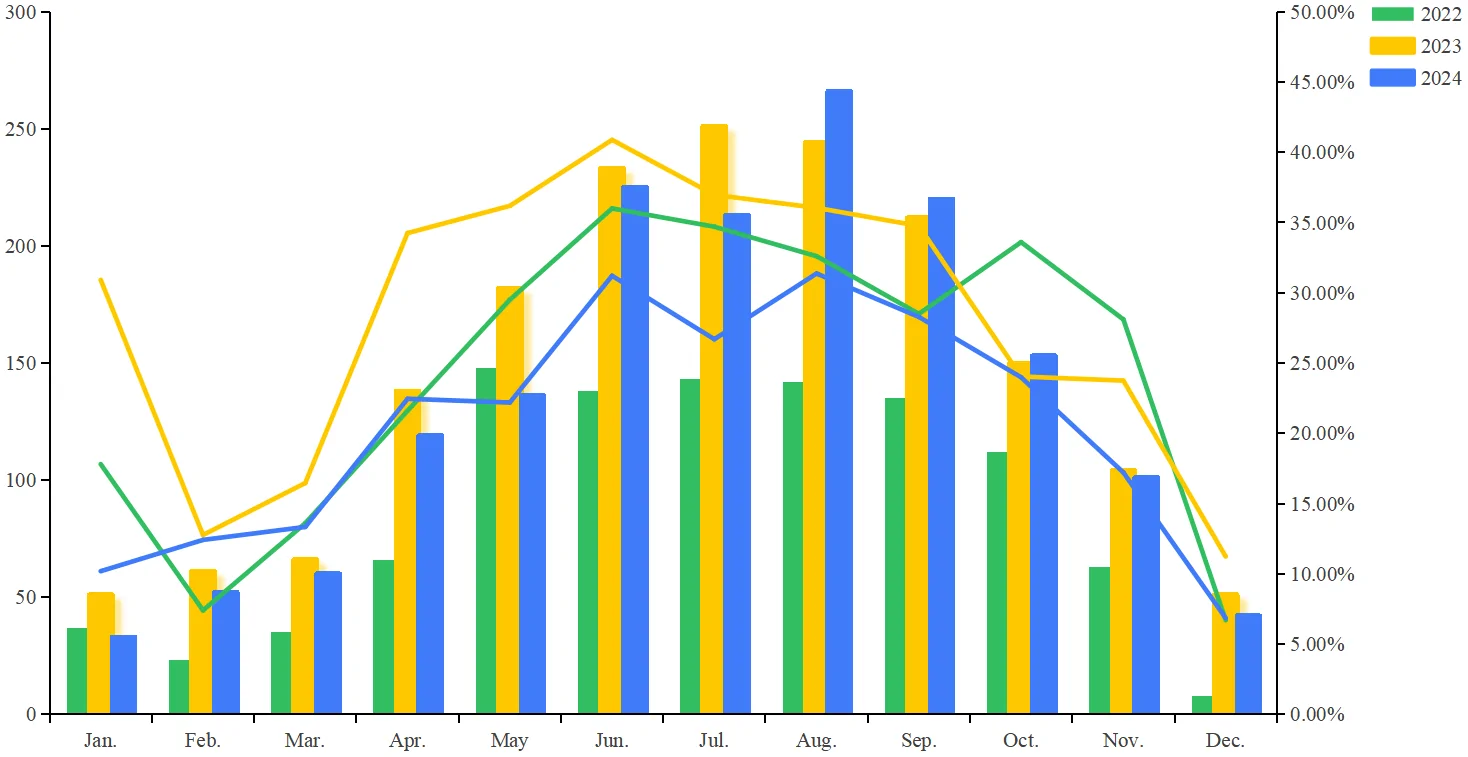
Monthly distribution and detection rates of *Salmonella enterica* in Guangdong, 2022–2024. The left Y-axis shows the total number of *Salmonella* isolates recovered in each month, represented by bars. The right Y-axis shows the monthly detection rate, represented by lines and calculated as the number of *Salmonella*-positive samples divided by the total number of samples tested in the corresponding month.

### 3.2 Antibiotic resistance profile

Antimicrobial susceptibility testing was performed on 1084 selected *Salmonella* isolates. As shown in **Table 1**, high susceptibility rates were observed for imipenem (97.79%, 1060/1084), cefoxitin (90.87%, 985/1084), gentamicin (85.52%, 927/1084), ceftazidime (78.78%, 854/1084), cefotaxime (73.34%, 795/1084) and trimethoprim-sulfamethoxazole (61.72%, 669/1084). In contrast, susceptibility to ciprofloxacin was low (38.47%, 417/1,084). The distribution of ciprofloxacin susceptibility by MDR status is shown in **Figure 2**. According to the definition of multidrug resistance, 771 isolates were classified as MDR and 313 as non-MDR, accounting for 71.13% and 28.87%, respectively. Resistance to ciprofloxacin was observed in both MDR and non-MDR isolates. Resistance to cefotaxime was also detected in both groups. In contrast, resistance to imipenem, cefoxitin, and gentamicin was rare among non-MDR isolates. The most common resistance profile was AMP-SAM-TCY-CHL-SXT (10.52%, 114/1,084), followed by AMP-TCY-CHL (6.00%, 65/1,084).

**Table 1:** Antimicrobial susceptibility profiles of 1084 Salmonella isolates .

Antibiotic categories	Antibiotic agents	Number of isolates (percentage)		
		Susceptible	Intermediate	Resistant
Quinolones	Ciprofloxacin (CIP)	417(38.47)	562(51.85)	105(9.69)
Aminopenicillins	Ampicillin (AMP)	246(22.69)	15(1.38)	823(75.92)
β-lactam/β-lactamase inhibitor combinations	Ampicillin-sulbactam (SAM)	463(42.71)	84(7.75)	537(49.54)
Cephems	Ceftazidime (CAZ)	854(78.78)	58(5.35)	172(15.87)
	Cefotaxime (CTX)	795(73.34)	14(1.29)	275(25.37)
	Cefoxitin (FOX)	985(90.87)	28(2.58)	71(6.55)
	Cefazolin (CZO)	310(28.60)	347(32.01)	427(39.39)
Tetracyclines	Tetracycline (TCY)	283(26.11)	23(2.12)	778(71.77)
Carbapenems	Imipenem (IPM)	1060(97.79)	12(1.11)	12(1.11)
Polypeptide antibiotics	Polymyxin B (POL)	679(62.64)	387(35.70)	18(1.66)
Aminoglycosides	Gentamicin (GEN)	927(85.52)	28(2.58)	129(11.90)
Phenicols	Chloramphenicol (CHL)	448(41.33)	13(1.20)	623(57.47)
Folate pathway inhibitors	Trimethoprim-sulfamethoxazole (SXT)	669(61.72)	0(0.00)	415(38.28)

Data are presented as no. (%) or as otherwise indicated.

**Figure 2:**
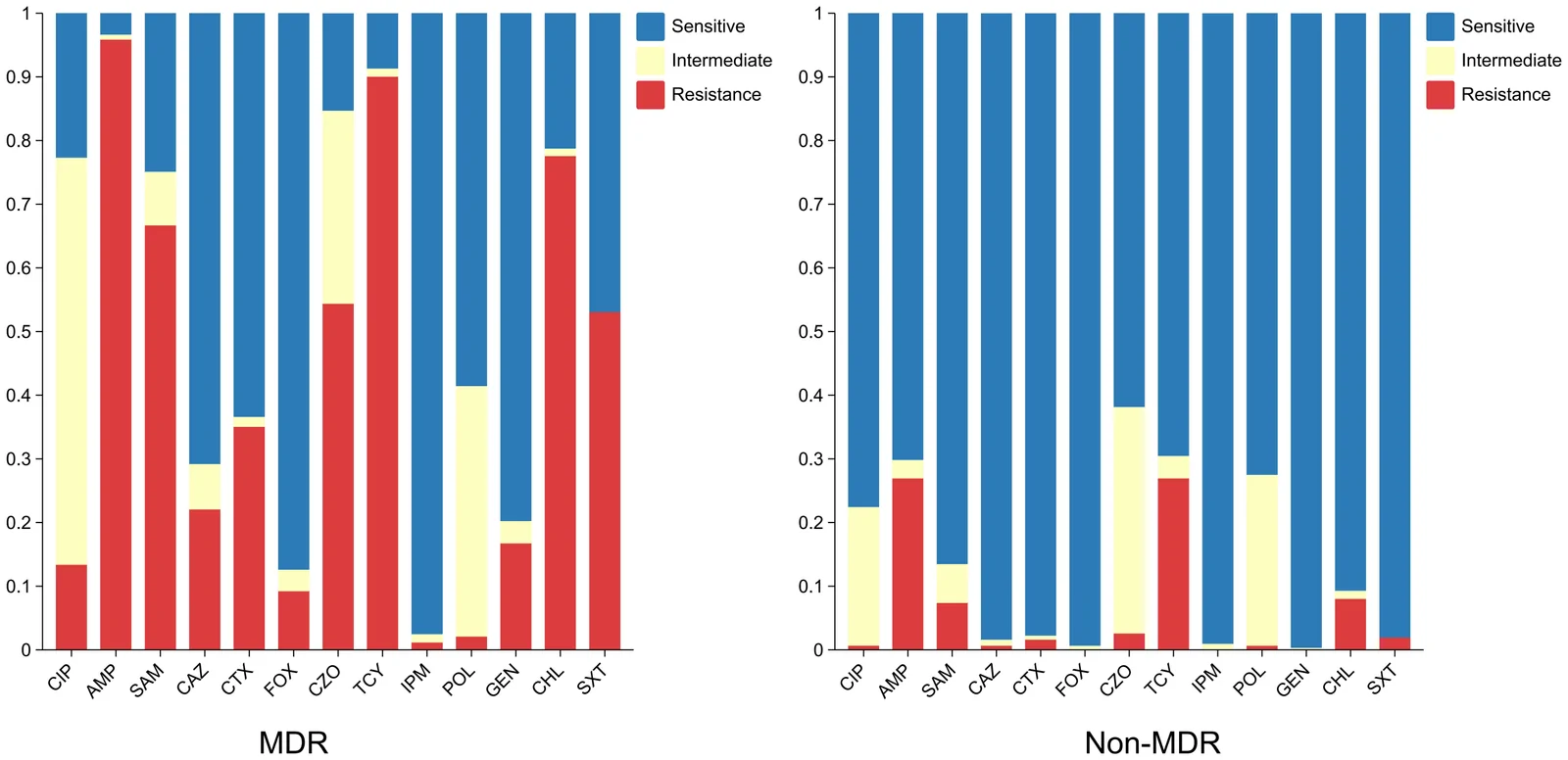
Antimicrobial susceptibility of MDR and non-MDR *Salmonella enterica* isolates. The y-axis represents the proportion of isolates in each susceptibility category, where 0 and 1 correspond to 0% and 100%, respectively.

### 3.3 Molecular characteristics of MDR and non-MDR *Salmonella enterica* isolates

Among the 170 Salmonella enterica isolates analyzed by both AST and WGS, CARD analysis identified 68 resistance-associated genes. After excluding chromosomally encoded intrinsic resistance determinants, ubiquitous intrinsic efflux components, regulatory genes, and biocide-associated determinants, 39 direct antimicrobial resistance genes were retained for comparative analysis and visualization in **Table 2** and **Figure 3**. Several other resistance genes, including *rmtB*, *ErmB*, *TEM-216*, *NDM-5*, *dfrA1*, and *FosA3*, were detected at low frequencies.

**Table 2:** Resistance genes of MDR and non- MDR *Salmonella enterica* isolates.

Resistance genes	All isolates (n=170)	MDR (n=105)	Non-MDR (n=65)	*P* value	CMH *P* value	FDR
Tetracycline						
*tet (A)*	64 (37.65)	51 (48.57)	13 (20.00)	**<0.001**	**<0.001**	**0.002**
*tet (B)*	38 (22.35)	29 (27.62)	9 (13.85)	**0.036**	0.686	0.686
*tet (M)*	24 (14.12)	22 (20.95)	2 (3.08)	**0.001**	**0.037**	0.095
Fluoroquinolone						
*qnrS1*	68 (40.00)	62 (59.05)	6 (9.23)	**<0.001**	**<0.001**	**<0.001**
*qnrB6*	8 (4.71)	7 (6.67)	1 (1.54)	0.156^a^	0.532	0.627
*aac (6* *’* *)-Ib-cr*	16 (9.41)	12 (11.43)	4 (6.15)	0.252	0.495	0.604
Aminoglycoside						
*aadA2 (ANT (3* *”* *))*	36 (21.18)	34 (32.38)	2 (3.08)	**<0.001**	**<0.001**	**<0.001**
*aadA (ANT (3* *”* *))*	35 (20.59)	31 (29.52)	4 (6.15)	**<0.001**	**<0.001**	**0.005**
*aadA23 (ANT (3* *”* *))*	7 (4.12)	7 (6.67)	0 (0.00)	**0.045** ^a^	0.220	0.330
*aac (6* *’* *)-Ib7*	10 (5.88) 0)	8 (7.62)	2 (3.08)	0.321^a^	0.575	0.627
*aph (3* *’’* *)-Ib*	67 (39.41) 0)	55 (52.38)	12 (18.46)	**<0.001**	0.109	0.190
*aph (6)-Id*	65 (38.24) 0)	52 (49.52)	13 (20.00)	**<0.001**	0.343	0.484
*aac (3)-IId*	21 (12.35) 0)	21 (20.00)	0 (0.00)	**<0.001**	**0.017**	**0.048**
*aac (3)-IV*	9 (5.29) 0)	8 (7.62)	1 (1.54)	0.156^a^	0.575	0.627
*aph (3* *’* *)-Ia*	15 (8.82) 0)	14 (13.33)	1 (1.54)	**0.008**	0.347	0.484
*aph (4)-Ia*	8 (4.71) 0)	7 (6.67)	1 (1.54)	0.156^a^	0.639	0.656
*rmtB*	1 (0.59) 0)	1 (0.95)	0 (0.00)	1.000^a^	-	-
*ANT (3* *’’* *)-IIa*	6 (3.53) 0)	6 (5.71)	0 (0.00)	0.083^a^	0.147	0.238
Beta-lactam						
*CTX-M-55*	26 (15.29)	26 (24.76)	0 (0.00)	**<0.001**	**0.003**	**0.013**
*CTX-M-* *27*	1 (0.59)	1 (0.95)	0 (0.00)	**1.000** ^a^	0.595	0.627
*CTX-M-65*	4 (2.35)	4 (3.81)	0 (0.00)	0.299^a^	0.448	0.564
*CTX-M-14*	6 (3.53)	6 (5.71)	0 (0.00)	0.083^a^	0.109	0.190
*TEM-1*	85 (50.00)	71 (67.62)	14 (21.54)	**<0.001**	**0.003**	**0.013**
*TEM-216*	2 (1.18) 0)	2 (1.90)	0 (0.00)	0.525^a^	0.448	0.564
*CMY-2*	6 (3.53) 0)	6 (5.71)	0 (0.00)	0.083^a^	0.189	0.295
*NDM-5*	2 (1.18) 0)	2 (1.90)	0 (0.00)	0.525^a^	0.448	0.564
Macrolides/Lincosamides						
*ErmB*	1 (0.59)	1 (0.95)	0 (0.00)	1.000^a^	0.595	0.627
*mphA*	19 (11.18)	19 (18.10)	0 (0.00)	**<0.001**	**0.017**	**0.048**
*linG*	12 (7.06)	12 (11.43)	0 (0.00)	**0.004** ^a^	0.112	0.190
Rifamycins						
*arr-2*	15 (8.82)	15 (14.29)	0 (0.00)	**0.001**	**0.040**	0.098
Phenicol						
*floR*	90 (52.94)	81 (77.14)	9 (13.85)	**<0.001**	**<0.001**	**<0.001**
*cmlA1*	27 (15.88)	26 (24.76)	1 (1.54)	**<0.001**	**0.002**	**0.010**
*cmlA5*	7 (4.12)	7 (6.67)	0 (0.00)	**0.045** ^a^	0.097	0.188
Sulfonamides						
*sul2*	81 (47.65)	68 (64.76)	13 (20.00)	**<0.001**	**0.011**	**0.039**
*sul3*	42 (24.71)	39 (37.14)	3 (4.62)	**<0.001**	**<0.001**	**<0.001**
Trimethoprim						
*dfrA12*	34 (20.00) 0)	31 (29.52)	3 (4.62)	**<0.001**	**<0.001**	**0.001**
*dfrA14*	30 (17.65) 0)	28 (26.67)	2 (3.08)	**<0.001**	**0.015**	**0.048**
*dfrA1*	2 (1.18) 0)	2 (1.90)	0 (0.00)	0.525^a^	-	-
Fosfomycin						
*FosA3*	2 (1.18) 0)	2 (1.90)	0 (0.00)	0.525^a^	0.083	0.171

Data are presented as no. (%) or as otherwise indicated. Boldface indicates statistical significance.

^a^ Estimated using Fisher’s exact test.

ST-stratified analyses were performed using the Cochran–Mantel–Haenszel test, with Benjamini–Hochberg FDR correction.

–, not estimable; MDR, multidrug resistance; ST, sequence type; FDR, false discovery rate.

**Figure 3:**
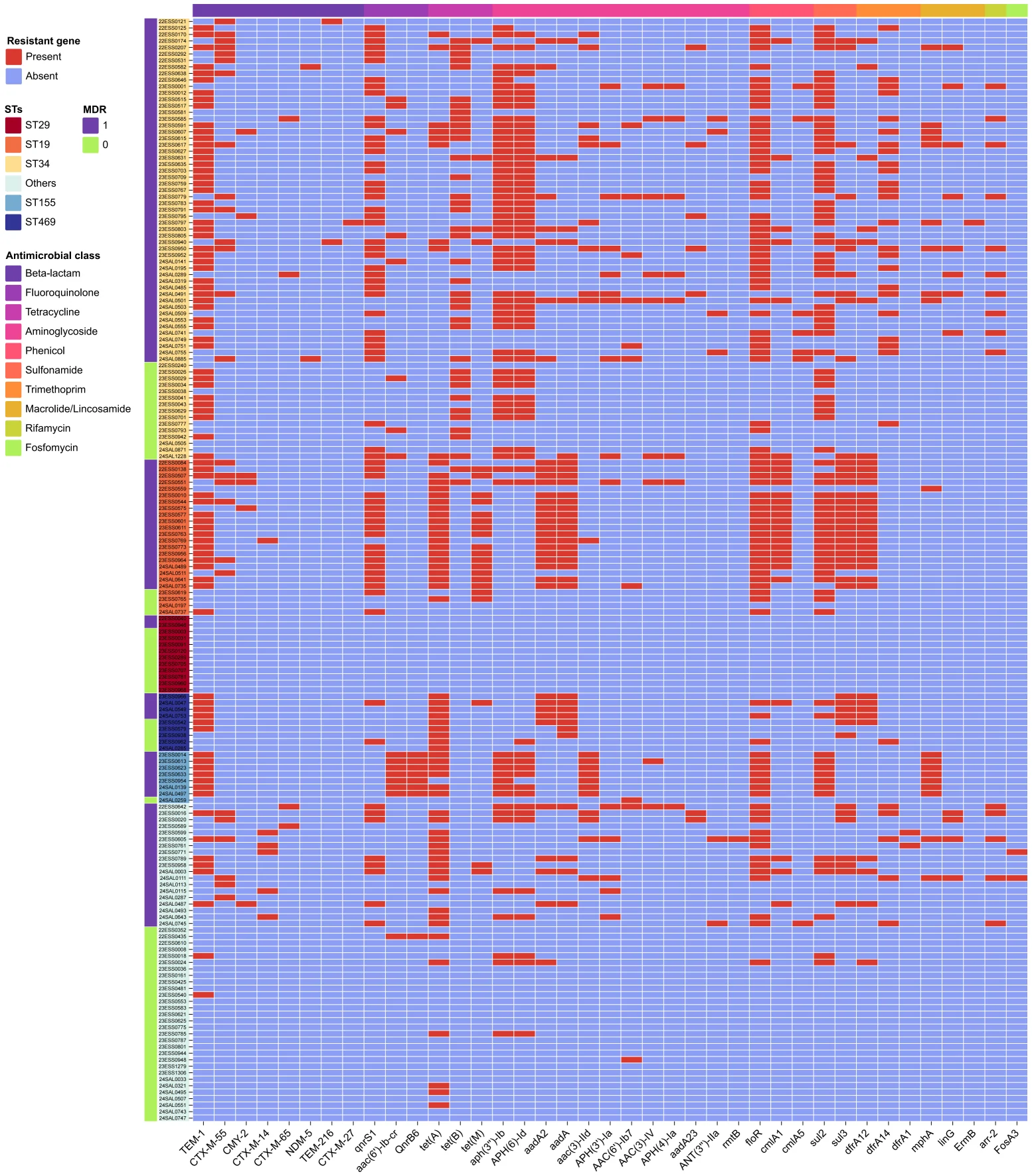
Resistance genes of MDR and non-MDR *Salmonella enterica* isolates. Red and blue indicate gene presence and absence, respectively. Genes were grouped by antimicrobial class, and isolates were arranged by sequence type, followed by MDR status and isolate identification number. The annotation bars indicate sequence type and MDR status.

In the unadjusted analyses, significant differences were observed in the distribution of multiple resistance genes between MDR and non-MDR isolates (**Table 2****).** Tetracycline resistance genes were more common in MDR isolates, including *tet(A)* (48.57% vs. 20.00%, P < 0.001), *tet(B)* (27.62% vs. 13.85%, P = 0.036), and *tet(M)* (20.95% vs. 3.08%, P = 0.001). The fluoroquinolone resistance gene *qnrS1* was also more frequent in MDR isolates (59.05% vs. 9.23%, P < 0.001). Significant associations with the MDR phenotype were also observed for the *β*-lactamase genes *CTX-M-55* (24.76% vs. 0.00%; P < 0.001) and *TEM-1* (67.62% vs. 21.54%; P < 0.001); the macrolide/lincosamide resistance genes *mphA* (18.10% vs. 0.00%; P < 0.001) and *linG* (11.43% vs. 0.00%; P = 0.004); and the rifamycin resistance gene *arr-2* (14.29% vs. 0.00%; P = 0.001). Furthermore, phenicol resistance genes (*floR*: 77.14% vs. 13.85%, *P* < 0.001; *cmlA1*: 24.76% vs. 1.54%, *P* < 0.001; *cmlA5*: 6.67% vs. 0.00%, *P* = 0.045), sulfonamide resistance genes (*sul2*: 64.76% vs. 20.00%, *P* < 0.001; *sul3*: 37.14% vs. 4.62%, *P* < 0.001), and trimethoprim resistance genes (*dfrA12*: 29.52% vs. 4.62%, *P* < 0.001; *dfrA14*: 26.67% vs. 3.08%, *P* < 0.001) were significantly more prevalent in MDR isolates. After stratification by sequence type, 14 gene–MDR associations, including *CTX-M-55*, *qnrS1*, and *floR*, remained significant after Benjamini–Hochberg correction (**Table 2**).

A total of 97 virulence genes were identified (**Figure 4**). These genes were mainly involved in fimbrial adherence, non-fimbrial adherence, secretion systems, macrophage inducible functions, magnesium uptake, regulation, stress adaptation, and toxin production. The most prevalent virulence genes were *sinH* (95.88%), *misL* (95.29%), *mig-14* (92.94%), *pagN* (92.35%), and *rpoS* (90.59%), followed by *siiE* (89.41%). Among MDR isolates, sinH and misL were detected in 96.19% and 95.24% of isolates, respectively. By contrast, *stfE* and *slrP* were less common in MDR isolates than in non-MDR isolates (14.29% vs. 41.54% and 24.76% vs. 56.92%, respectively).

**Figure 4:**
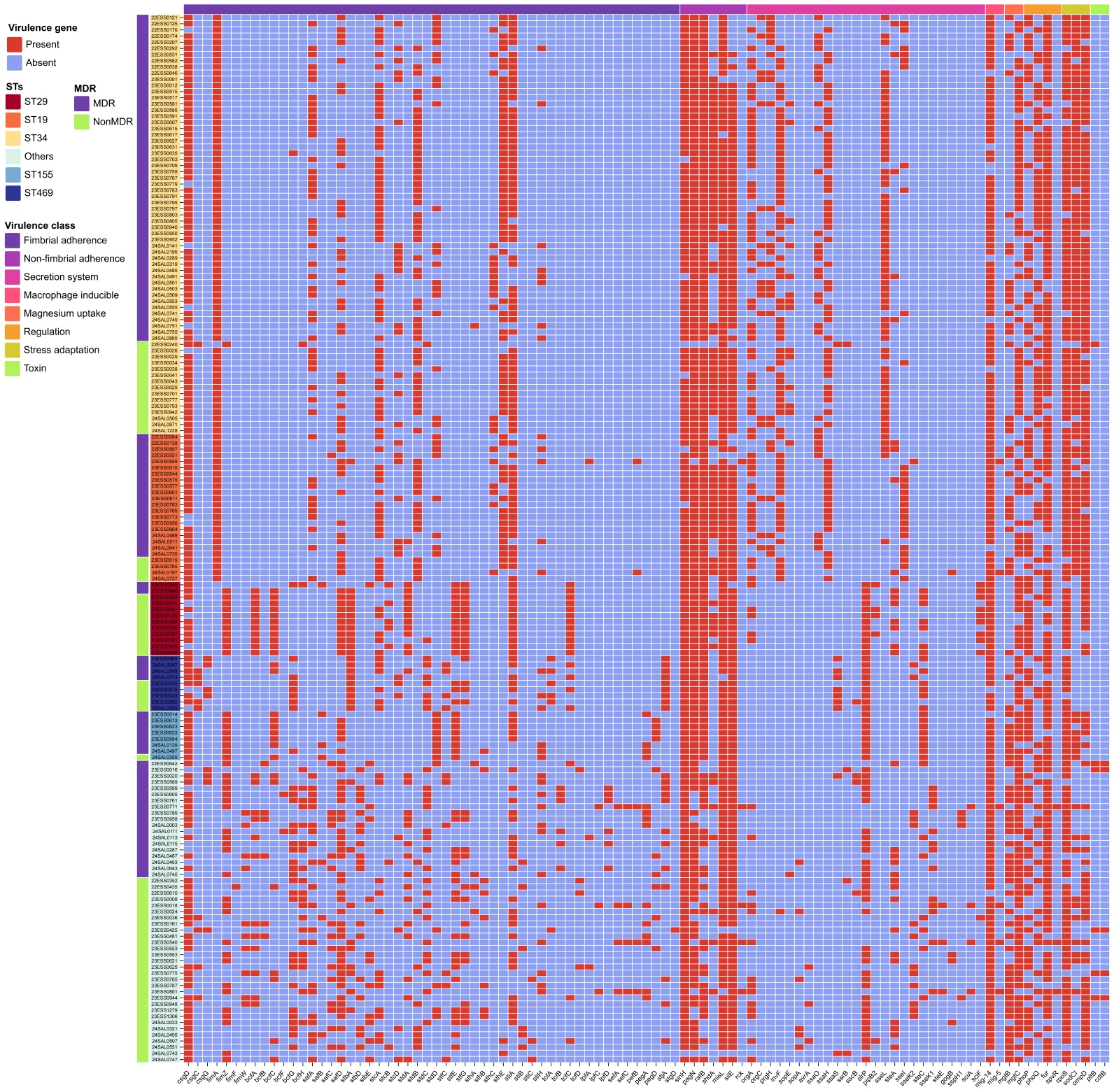
Virulence genes of MDR and non-MDR *Salmonella enterica* isolates. Red and blue indicate gene presence and absence, respectively. Genes were grouped by virulence class, and isolates were arranged by sequence type, followed by MDR status and isolate identification number. The annotation bars indicate sequence type and MDR status.

### 3.4 *Salmonella* serovars, sequence typing and phylogenetic analysis

A total of 61 distinct serovars were identified. *S.* I 4,[5],12:i:- was the most common, accounting for 46.31% (502/1084). It was followed by *S.* Typhimurium (19.19%, 208/1084), *S.* Enteritidis (5.81%, 63/1084), and *S.* Stanley (5.35%, 58/1084). The nine predominant serovars shown in **Table 3** accounted for 89.48% (970/1084) of the isolates. The remaining serovars accounted for 10.52% (114/1084), and their detailed distribution is provided in **Supplementary Table S3**. Comparisons between MDR and non-MDR isolates showed significant differences in serovar distribution (**Table 3**). *S.* I 4,[5],12:i:- (55.51% vs. 23.64%, P < 0.001) and *S.* Typhimurium (22.31% vs. 11.50%, P < 0.001) were more common in MDR isolates. By contrast, *S.* Enteritidis (4.02% vs. 10.22%, P < 0.001), *S.* Stanley (1.69% vs. 14.38%, P < 0.001), and *S.* Braenderup (0.00% vs. 9.90%, P < 0.001) were less common in MDR isolates. Whole-genome sequencing was performed on 170 *Salmonella* enterica isolates. Based on phenotypic serotyping, these 170 isolates belonged to 29 serovars (**Figure 5**). The most common serovar was *S.* I 4,[5],12:i:- (37.06%, 63/170), followed by *S.* Typhimurium (18.24%, 31/170), *S.* Stanley (8.24%, 14/170), *S.* Rissen (5.29%, 9/170), *S.* London (4.12%, 7/170), *S.* Kentucky (3.53%, 6/170), *S.* Weltevreden (2.94%, 5/170), and *S.* Derby (2.94%, 5/170). Sequence typing identified 29 sequence types (STs) among the 170 isolates. The most common STs were ST34 (40.00%, 68/170) and ST19 (14.12%, 24/170), followed by ST29 (7.06%, 12/170), ST469 (5.29%, 9/170), and ST155 (4.71%, 8/170). Comparisons between MDR and non-MDR isolates showed significant differences in the distribution of ST34, ST19, and ST29 (all P < 0.05; **Table 4**). ST34 and ST19 were more common in MDR isolates. *S.* I 4,[5],12:i:- was mainly associated with ST34, *S.* Typhimurium with ST19, *S*. Stanley with ST29, *S*. Rissen with ST469, and *S*. London with ST155. In the phylogenetic tree, isolates belonging to S. I 4,[5],12:i:- and S. Typhimurium showed relatively close genetic relationships.

**Table:** Table 3 Serovar identification of MDR and non-MDR *Salmonella* isolates .

Serovar identification	*Salmonella* isolates (n=1084)	MDR isolates (n=771)	Non-MDR isolates (n=313)	*P* value
*S*.I 4,[5],12:i:-	502 (46.31)	428 (55.51)	74 (23.64)	**<0.001**
*S*.Typhimurium	208 (19.19)	172 (22.31)	36 (11.50)	**<0.001**
*S*.Enteritidis	63 (5.81)	31 (4.02)	32 (10.22)	**<0.001**
*S*.Stanley	58 (5.35)	13 (1.69)	45 (14.38)	**<0.001**
*S*.Braenderup	31 (2.86)	0 (0.00)	31 (9.90)	**<0.001**
*S*.Rissen	34 (3.14)	24 (3.11)	10 (3.19)	0.944
*S*.London	30 (2.77)	25 (3.24)	5 (1.60)	0.135
*S*.Derby	24 (2.21)	12 (1.56)	12 (3.83)	0.021
*S*.Goldcoast	20 (1.85)	13 (1.69)	7 (2.24)	0.542

Data are presented as no. (%) or as otherwise indicated. Boldface indicates statistical significance.

MDR, Multidrug Resistance.

**Figure 5:**
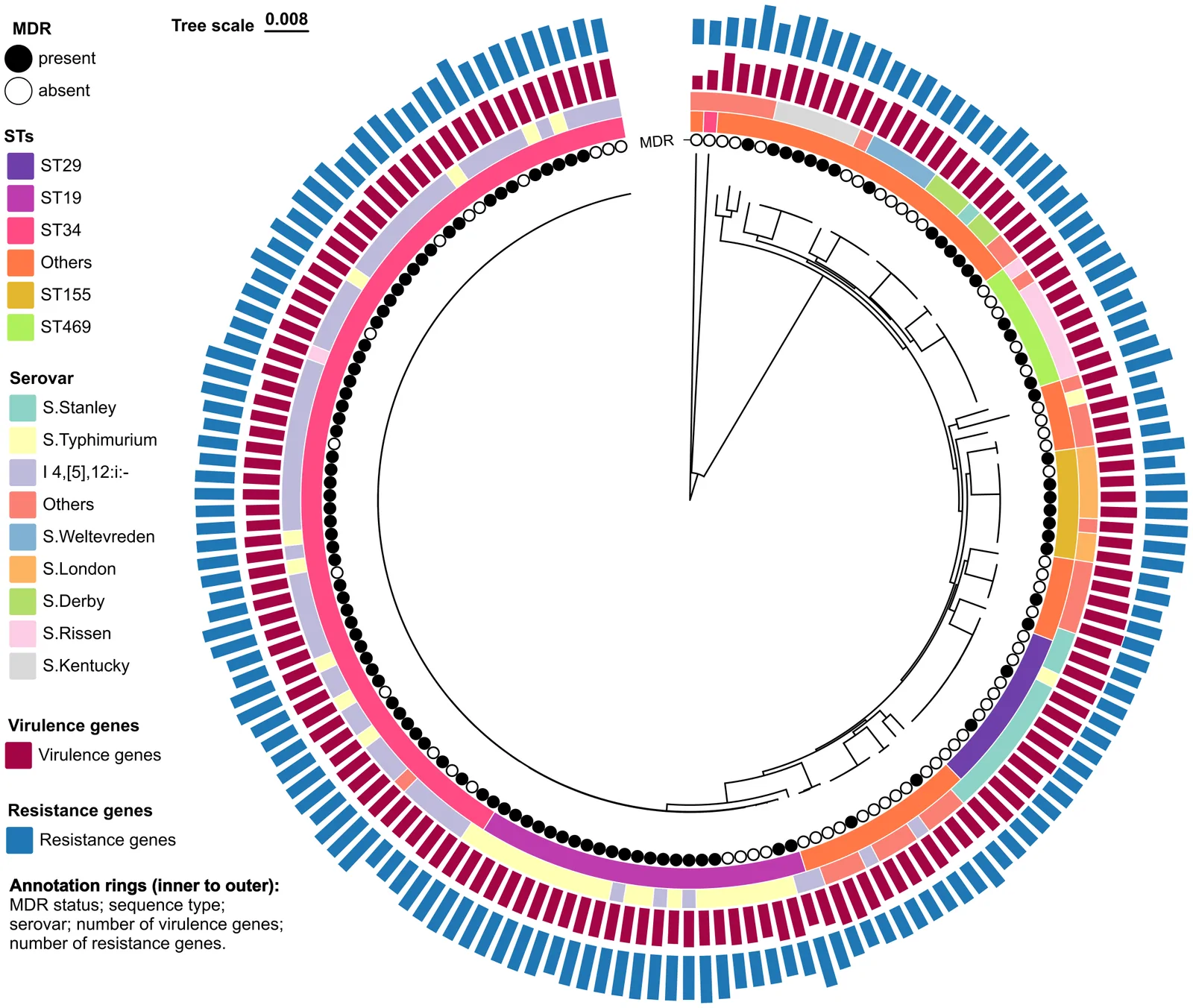
Midpoint-rooted maximum-likelihood phylogenetic tree of 170 *Salmonella* enterica isolates. The concentric annotation rings, from the inside to the outside, represent MDR status, sequence type, phenotypic serovar, the number of virulence genes, and the number of resistance genes. Branch lengths are proportional to nucleotide substitutions per site, and the scale bar indicates 0.008 substitutions per site. Branch support was assessed using 1000 ultrafast bootstrap replicates; bootstrap values were not displayed in the figure to maintain visual clarity because of the large number of isolates and annotations.

**Table 4:** Predominant genotypes of MDR and non-MDR *Salmonella* isolates .

Genotypes	*Salmonella* isolates (n=170)	MDR isolates (n=105)	Non-MDR isolates (n=65)	*P* value
ST34	68(40.00)	53(50.48)	15(23.08)	**<0.001**
ST19	24(14.12)	20(19.05)	4(6.15)	**0.019**
ST29	12(7.06)	2(1.90)	10(15.38)	**0.001** ^a^
ST469	9(5.29)	4(3.81)	5(7.69)	0.305^a^
ST155	8(4.71)	7(6.67)	1(1.54)	0.156^a^

Data are presented as no. (%) or as otherwise indicated. Boldface indicates statistical significance.

^a^ Estimated using Fisher’s exact test.

MDR, Multidrug Resistance.

### 3.5 Machine-learning-assisted prioritization of candidate genomic features associated with MDR

Machine-learning analyses were performed to prioritize candidate genomic features associated with the MDR phenotype. A combined strategy using XGBoost and LASSO regression was applied to rank and prioritize candidate features. In the XGBoost analysis, *floR* was the highest-ranked feature, whereas *CTX-M-55* was also ranked among the important features associated with MDR. LASSO selected 16 features, including 10 resistance genes and 6 virulence genes. Among the LASSO-selected features, *TEM-1*, *floR*, and *CTX-M-55* showed the strongest associations with the MDR phenotype. Comparison of the XGBoost and LASSO results identified 10 overlapping genes (**Figure 6A**). Both methods consistently prioritized *floR* and *CTX-M-55* as important features associated with the MDR phenotype. **Figure 6B** shows the feature importance ranking of the overlapping genes, and **Figure 6C** shows the prevalence of the 10 shared genes in MDR and non-MDR isolates.

**Figure 6:**
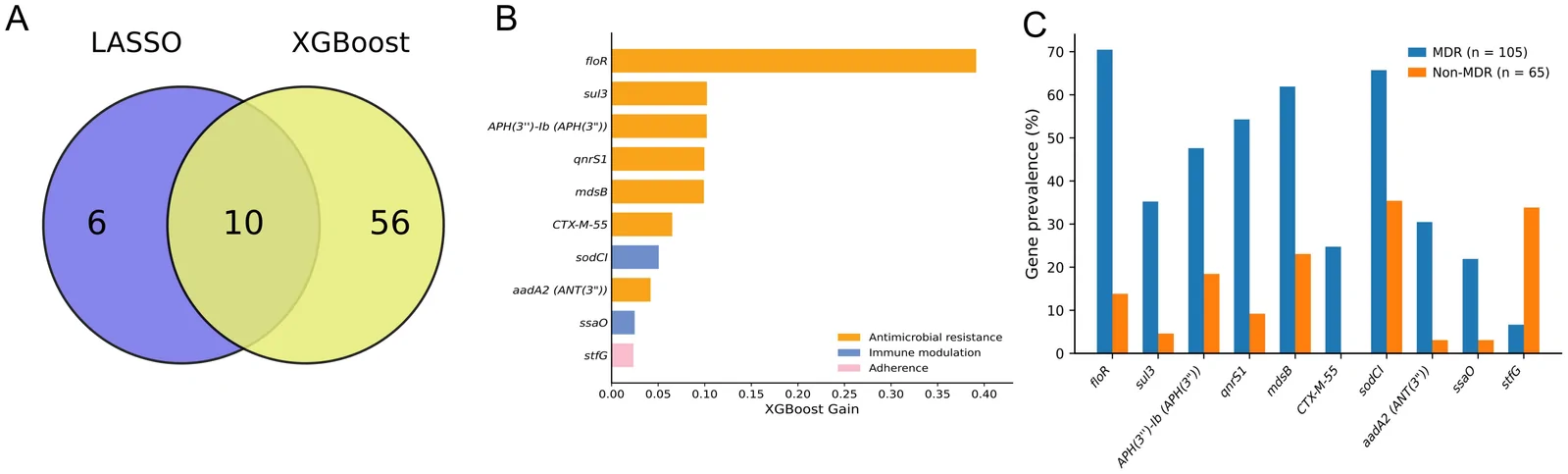
Machine learning–based prioritization of candidate genetic features associated with the multidrug-resistant (MDR) phenotype. **(A)** Venn diagram showing the overlap between genes selected by the LASSO and XGBoost analyses. Ten genes were identified by both methods. **(B)** XGBoost Gain values of the 10 overlapping genes, ranked from highest to lowest. Colors indicate the functional categories of the genes. **(C)** Distribution of the 10 shared genes in MDR and non-MDR Salmonella isolates.

## 4 Discussion

In this study, we found a high prevalence of MDR among clinical *S. enterica* isolates in Guangdong. Most isolates remained susceptible to imipenem. However, susceptibility to ciprofloxacin was low, suggesting substantial fluoroquinolone resistance. This pattern is consistent with recent surveillance from China, which also found a high MDR burden in *Salmonella* isolates (Yu et al., 2024; Kong et al., 2025; Wang et al., 2023). MDR isolates more often carried *CTX-M-55*, *qnrS1*, and *TEM-1*. This may help explain the lower susceptibility to cephalosporins and fluoroquinolones in our dataset. Similar patterns have also been reported in *Salmonella* isolates from China (Li et al., 2022; Lv et al., 2025). These findings add regional data on *Salmonella* resistance in Guangdong.

We observed a clear seasonal pattern in *Salmonella* isolation, with higher numbers from May to October and a peak in June. This pattern is consistent with previous reports from China and other regions, which also showed a summer or early autumn peak in *Salmonella* infections (Wang et al., 2025). It may be related to warmer temperatures, which favor bacterial growth and increase the risk of food contamination (Damtew et al., 2024; Anikeeva et al., 2024).

*S*. I 4,[5],12:i:- and *S.* Typhimurium were the dominant serovars in our study, and *S*. I 4,[5],12:i:- was the most common. Some discrepancies were observed between phenotypic serotyping and SeqSero2 prediction, particularly in distinguishing *S*. Typhimurium from its monophasic variant *S.* I 4,[5],12:i:-. Similar discrepancies have been reported previously, as genome-based serotyping may identify second-phase flagellar determinants even when the corresponding antigen is not phenotypically expressed, potentially resulting in the prediction of *S.* Typhimurium for phenotypically monophasic isolates (Banerji et al., 2020; Arrieta-Gisasola et al., 2020). The predominance of these serovars is consistent with recent reports from China and other regions (Yu et al., 2024; Su et al., 2026). MDR isolates were mainly distributed in these two serovars. ST34 and ST19 were the predominant sequence types among MDR isolates, consistent with previous reports (Ingle et al., 2021; Chung The et al., 2023).

In our study, MDR isolates carried more resistance genes than non-MDR isolates. In particular, *CTX-M-55*, *qnrS1*, and *TEM-1* were more common in the MDR group. Similar findings have been reported in clinical and food-associated *Salmonella* isolates from other regions of China, suggesting that this pattern is not unique to our dataset (Yu et al., 2024; Kong et al., 2025; Lv et al., 2025; Cao et al., 2025). This pattern may be related to long-term antimicrobial selection pressure, under which genes such as *CTX-M-55* and *qnrS1* may be more likely to persist in MDR isolates (Jiang et al., 2024; Zeng et al., 2022). In addition, these genes may spread together under shared selection pressure. Previous genomic studies have shown that *CTX-M-55* and *qnrS1* are often found on mobile genetic elements or plasmids such as IncHI2 (Li et al., 2022; Lv et al., 2025; Mai et al., 2025). This may help several resistance genes spread together. Our earlier results also showed that MDR isolates were mainly found in specific serovars and sequence types. This suggests that the enrichment of these genes may also be related to the expansion of dominant clones. In particular, ST34-related resistant lineages have shown ongoing expansion and regional spread (Chung The et al., 2023; Sun et al., 2024, 2025). These findings suggest that clinical MDR *Salmonella* in Guangdong may be associated with key resistance gene combinations and expansion of dominant clones. Although 14 gene-MDR associations remained significant after ST-stratified analysis, residual confounding by clonal population structure cannot be completely excluded. These genes should therefore be interpreted as MDR-associated features rather than as independent determinants of MDR.

Virulence gene profiles differed between MDR and non-MDR isolates. Genes such as *sinH*, *pagN*, and *misL* were more common in the MDR group, whereas *stfE* and *slrP* were more common in the non-MDR group. Similar differences have also been reported in other *Salmonella* studies, and these differences were often related to serovar, sequence type, or lineage background rather than a simple increase in overall virulence (Cheng et al., 2019; Sedrakyan et al., 2022; Guo et al., 2023; Sodagari and Habib, 2025). In our dataset, MDR isolates were mainly found in specific serovars and sequence types. This suggests that the enrichment of *sinH*, *pagN*, and *misL* in the MDR group may partly reflect the expansion of dominant lineages (Sun et al., 2024, 2025; Liao et al., 2025; Chung The et al., 2023). Previous studies also indicate that *pagN* is involved in adhesion and invasion, *misL* contributes to intestinal colonization, and *sinH* is linked to host interaction, which may support colonization and persistence of MDR-associated lineages (Dorsey et al., 2005; Lambert and Smith, 2008; Srednik et al., 2023). Overall, our findings suggest that clinical MDR *Salmonella* in Guangdong differs from non-MDR isolates not only in resistance genes, but also in virulence genes. The machine learning results were consistent with the earlier analysis. Both XGBoost and LASSO identified *floR* and *CTX-M-55* as important features associated with MDR. Among them, *CTX-M-55* is closely linked to third-generation cephalosporin resistance and has been repeatedly reported in ST34 lineages in China (Bao et al., 2024; Zhang et al., 2022). By contrast, *floR* may reflect a multidrug resistance background because it is often found with other resistance genes in MDR regions or gene clusters on plasmids (Mai et al., 2025). The analyses also selected several virulence genes. This suggests that the difference between MDR and non-MDR isolates may reflect not only resistance genes, but also the genetic background of dominant lineages (Liu et al., 2025; de Sousa Violante et al., 2024).

This study has some limitations. First, all isolates came from one province in China, which may limit the generalizability of the findings to other regions. Second, although the WGS subset included isolates from different years, regions, serovars, and MDR categories, proportional allocation was not used in selecting isolates for sequencing. Future studies using stratified random sampling with proportional allocation may further strengthen representativeness. Third, residual confounding related to clonal population structure may remain despite the ST-stratified analysis. Finally, only clinical isolates were included, precluding direct comparison with isolates from food, animals, and the environment.

## 5 Conclusion

In summary, clinical *S. enterica* isolates from Guangdong showed a high prevalence of MDR. MDR isolates were mainly associated with dominant lineages such as ST34 and ST19 and with key resistance genes including *CTX-M-55*, *qnrS1*, and *floR*. These findings add regional data on the genomic features of MDR *Salmonella* in Guangdong.

## Data Availability

The datasets presented in this study can be found in online repositories. The names of the repository/repositories and accession number(s) can be found below: https://www.ncbi.nlm.nih.gov/, PRJNA1544743.
